# Characterization of the Complete Mitochondrial Genome Sequence of the Globose Head Whiptail *Cetonurus globiceps* (Gadiformes: Macrouridae) and Its Phylogenetic Analysis

**DOI:** 10.1371/journal.pone.0153666

**Published:** 2016-04-19

**Authors:** Xiaofeng Shi, Peng Tian, Rongcheng Lin, Dingyong Huang, Jianjia Wang

**Affiliations:** 1 Laboratory of Marine Biology and Ecology, Third Institute of Oceanography, State Oceanic Administration, Xiamen, P.R. China; 2 Ocean College, Zhejiang University Hangzhou, P.R. China; Sichuan University, CHINA

## Abstract

The particular environmental characteristics of deep water such as its immense scale and high pressure systems, presents technological problems that have prevented research to broaden our knowledge of deep-sea fish. Here, we described the mitogenome sequence of a deep-sea fish, *Cetonurus globiceps*. The genome is 17,137 bp in length, with a standard set of 22 transfer RNA genes (tRNAs), two ribosomal RNA genes, 13 protein-coding genes, and two typical non-coding control regions. Additionally, a 70bp tRNA^Thr^-tRNA^Pro^ intergenic spacer is present. The *C*. *globiceps* mitogenome exhibited strand-specific asymmetry in nucleotide composition. The AT-skew and GC-skew values in the whole genome of *C*. *globiceps* were 0 and -0.2877, respectively, revealing that the H-strand had equal amounts of A and T and that the overall nucleotide composition was C skewed. All of the tRNA genes could be folded into cloverleaf secondary structures, while the secondary structure of tRNA^Ser(AGY)^ lacked a discernible dihydrouridine stem. By comparing this genome sequence with the recognition sites in teleost species, several conserved sequence blocks were identified in the control region. However, the GTGGG-box, the typical characteristic of conserved sequence block E (CSB-E), was absent. Notably, tandem repeats were identified in the 3' portion of the control region. No similar repetitive motifs are present in most of other gadiform species. Phylogenetic analysis based on 12 protein coding genes provided strong support that *C*. *globiceps* was the most derived in the clade. Some relationships however, are in contrast with those presented in previous studies. This study enriches our knowledge of mitogenomes of the genus *Cetonurus* and provides valuable information on the evolution of Macrouridae mtDNA and deep-sea fish.

## Introduction

Deep-sea fish assemblages are an important component in bathyal and abyssal ecosystems. Nevertheless, the particular environmental characteristics of deep water such as its immense scale and high pressure present special technological problems that have largely blocked our knowledge of these fish. To date, the information on deep-water fish has been generally scarce and has focused on species of intertidal, coastal and near-coastal zones. However, as research has progressed, a number of deep-sea fish have been characterized as having slow growth and low fecundity [1, 2]. As a result, these fish are highly vulnerable to anthropogenic effects such as overfishing and have low resilience [3]. Increasing research interest has developed inconservation as well as in scientific and economic topics regarding deep-sea fish species [4, 5].

The macrourids (Pisces: Macrouridae), belonging to the gadiform order and also known as grenadiers or rattails, are one of the most important demersal species living at great depths on the continental slopes and the abyssal planes. They form a dominant part of the deep-sea fish fauna due to both the high number of species and their positive contribution to the global biomass of ecosystems. Among more than 300 species contained in the family, Macrouridae species are ubiquitous in the world’s oceans, from the Arctic to the Antarctic [6–8]. Such a wide latitudinal and bathymetric distribution makes them an ideal model for studying the mechanism of adaptation of organisms to the lower bathyal, abyssal and hadal depth zones [8]. Combining such data with fossil records would allow us to gain novel insights into possible early migration routes, evolution and adaptive radiation events of deep water demersal fish families. In recent years, some strategies to extensively explore the distribution patterns, biodiversity, feeding habits and life histories of such fish have been performed by many researchers [6, 8, 9]. However, until now, only preliminary investigations have been conducted regarding the genetic relationships, evolution and adaptation of these fish. Additionally, the morphology-based phylogenetic hypotheses regarding the family remain unclear and are disputed, indicating the requirement for more molecular data to clarify the relationships of these fish [10, 11].

Genomic information is considered to be reliable for the efficient implementation of strategies to study evolutionary relationships, phylogeography and phylogeny [12, 13]. There has been consistent affirmation of the value of mitochondrial DNA (mtDNA) as the second genome for corresponding studies because of its maternal inheritance, relatively fast evolutionary rate, high copy number and lack of intermolecular genetic recombination [14, 15]. Mitochondrial genomes have now redefined rules in genetics [16] and become the most important tool in studying hypotheses on evolution [17]. Moreover, they have initiated the dabate of the relationship between adaptive processes and organismal and genomic complexity [18]. Moreover, recent evidence gathered with genome synteny analysis has revealed a number of shared unique mitochondrial gene features in Gadiformes, supporting a further role of understanding the functions and evolution of Gadiformes [19, 20]. In this study, we assembled the mitochondrial genome of globose head whiptail, *Cetonurus globiceps*, which is generally distributed at a depth ranging from 740 m to 4621 m [21, 22]. To date, little is known regarding this species. Additionally, it is the first species of *Cetonurus* for which the mitogenome has been completely sequenced. Given the wide use of mitochondrial genome sequences in evolutionary and phylogenetic inferences, this study will provide valuable information on the complete genetic contents of the mitochondrial genome of *C*. *globiceps*. A comparative analysis of the gene order of this genome and other closely related species was performed to provide deeper insight into the evolution of Macrouridae mtDNA. All of the discoveries presented here will further allow us to better understand Macrouridae migration and will help us to gain more insight into the genesis and evolution of deep-sea species.

## Materials and Methods

### Sampling

The globose head whiptail was captured at a depth of 2,728 m in the deep-sea hydrothermal vent region of the south Mid-Atlantic Ridge (13°21′W, 15°10′S) during the 5th Leg of the 22 Cruise of the China Ocean Mineral Resources Research and Development Association (COMRA) onboard R/V Dayang Yihao, using a combined biological sampler designed by the State Key Laboratory of Exploitation and Utilization of Deep Sea Mineral Resources of China. Fieldwork including the collection of fish was completed in the publically available area (high seas) and no specific permissions were required for these locations and activities according to United Nations Convention on the Law of the Sea. The field studies did not involve endangered or protected species according to the IUCN Red List. Our study was conducted with the approval from the Institutional Animal Care and Use Committee at the Third institute of Oceanography, State Oceanic Administration. All operations were performed according to international guidelines concerning the care and treatment of experimental animals. Species identification was confirmed based on morphological features, and a small portion of the hepatic tissue was aseptically sampled and preserved in 95% ethanol until use.

### DNA Extraction, PCR Amplification and Sequencing

Total genomic DNA, which was used as a PCR template, was extracted from hepatic tissue samples using the DNeasy tissue kit (Qiagen, Germany), following the manufacturer’s protocol. A long PCR approach was performed to obtain the complete *C*. *globiceps* mitogenome sequence. Several homology-degenerate primer sets were designed based on aligned mitogenome sequences of *Lota lota* (GenBank: AP004412.1), *Merlangius merlangus* (GenBank: DQ020496.1), and *Ventrifossa garmani* (GenBank: AP008991.1) (S1 Table). All PCRs were conducted in a Biometra thermal cycler (Biometra, Germany). The cycling profile was designed to have an initial denaturation step at 95°C for 5 min, followed by 32 cycles of denaturation at 94°C for 30 s, annealing at 50°C for 30 s and elongation at 72°C for 3–6 min. The process was completed with a final elongation at 72°C for 10 min. A total reaction volume of 25μl included 20μl sterile deionized water, 2.5μl 10× LA PCR buffer (Mg^2+^ plus, TaKaRa), 0.5μl dNTP mix (10 mM each, TaKaRa), 0.5μl of each primer (10 μM), 0.5μl of 1 U/μl LA Taq DNA polymerase (TaKaRa, Japan) and 0.5μl DNA template (50 ng/μl). PCR products were confirmed visually on a 1.5% agarose gel (1×TAE) and purified with a MiniBEST Agarose Gel DNA Extraction Kit (TaKaRa, Japan). The purified product was then sequenced on an ABI Prism 3730 automated sequencer (Applied Biosystems, USA).

### Sequence Analysis

Target sequences were assembled with the program Seqman within Lasergene software [23], and the final complete sequence obtained was further checked manually. The location of the 13 protein-coding genes and two rRNAs were initially determined via use of the software program DOGMA [24] using the default settings. These were later refined via alignment to DNA and amino acid sequences of the mitochondrial genomes of other 10 Gadiform species shown in Table 1 using the program Clustal X, version 2.0 [25]. The majority of transfer RNA (tRNA) genes were recognized by tRNA Scan-SE 1.21 [26] using default parameters with a cut-off score of 2. The remaining tRNA genes were identified by their proposed structures. The non-coding regions were predicted by sequence homology with Clustal X, version 2.0. The potential secondary structures of origin of light strand replication (O_L_) and the tRNA^Thr^-tRNA^Pro^ intergenic spacer were analyzed with the mfold web server [27]. The tandem repeats in the control region (CR) were analyzed with Tandem Repeat Finder, Ver. 3.21 [28]. The complete mitochondrial genomic DNA sequence was deposited in the GenBank database (GenBank accession NO. KF751382).

The codon usage of the 13 protein-coding genes was summarized with MEGA 6 [29]. The AT-skew [(A−T)/(A+T)] and GC-skew [(G−C)/(G+C)] values were used to measure the nucleotide compositional differences between genes [30].

### Phylogenetic Analysis

To elucidate the phylogenetic position of *C*. *globiceps* within Gadiformes, other Gadiforme mitogenomes available in GenBank were obtained. *Sardinops melanostictus* (GenBank accession NO. NC_002616) was selected as the outgroup, with reference to the phylogenetic analysis of the ray-finned fish mitogenomic data [31, 32]. Every gene except *ND6* [19, 33] was translated into its amino acid sequence in MEGA 6and was aligned separately using Clustal X with default settings. All the 12 protein-coding gene alignments, excluding the stop codons were subsequently back-translated to the corresponding nucleotide sequences and concatenated into single multiple sequence alignments for analysis. Bayesian inference (BI), maximum likelihood (ML) and neighbor joining (NJ) were used for phylogenetic reconstructions [34–36].

The best model for the evolution of nucleotide substitution was determined by jModeltest [37]. According to the Akaike information criterion (AIC), HKY+I+G was selected as the best model for *ND1*, GTR+I+G was selected for *ND2* and *ND4*, TIM1+I+G was selected for *COI*, *COIII*, and *ND5*, TPM3uf+I+G was selected for *COII*, *ND4L* and *Cytb*, TVM+G was selected for *ATP8*, TrN+I+G was selected for *ATP6*, and TIM2+I+G was selected for *ND3*. A partitioned Bayesian phylogenetic analysis was performed using MrBayes version 3.1.2 with the Markov Chain Monte Carlo (MCMC) method [38]. Fifteen million generations with four chains were run, sampling every 500 generations and discarding 25% of the initial trees as burn-in. The standard deviation of split frequencies was below 0.01. Neighbor joining was carried out with the Dayhoff matrix model, implemented with MEGA 6. The consensus tree was obtained with 1000 bootstrap replicates. Partitioned maximum likelihood phylogenetic analysis was performed with GARLI v.0.951 [39]. Each analysis was run with 100 replicates using a random starting tree. Search replicates were evaluated by using likelihood scores, retaining only the replicate with the best score.

## Results and Discussion

### Genome Organization and Composition

**Fig 1:**
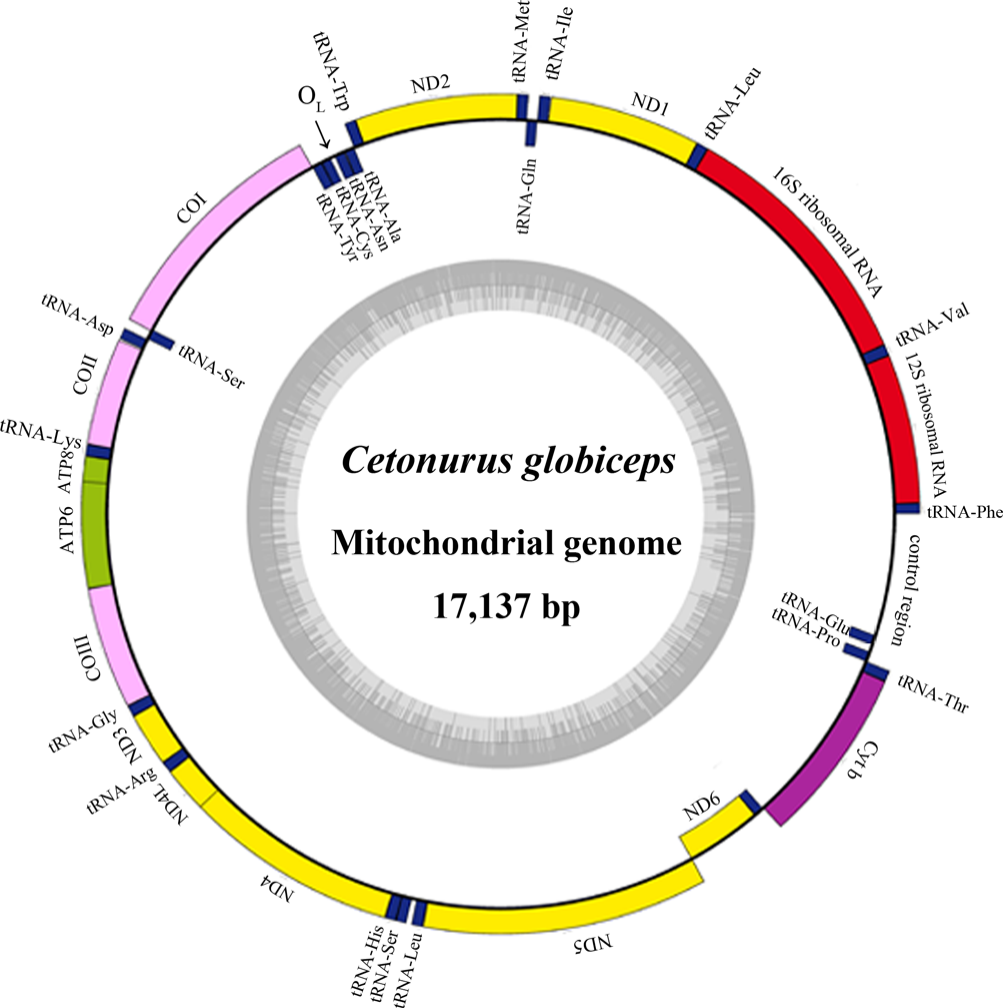
Gene map and organization of the complete mitochondrial genome of *C*. *globiceps*. Genes encoded on the heavy and light strand are shown outside and inside the circle, respectively. The inner grey ring indicates the GC content.

The complete mitochondrial DNA sequence (17,137bp in length) consists of 13 protein-coding genes, 22 tRNA genes, two ribosomal RNA genes (12S rRNA and 16S rRNA) and two major non-coding regions (the origin of light strand replication (O_L_) and control region (CR)) (GenBank accession NO. KF751382) (Fig 1). Additionally, a tRNA^Thr^-tRNA^Pro^ (T-P) intergenic spacer (70 bp in length) unique to Gadiformes was observed [20, 40]. The *C*. *globiceps* mitogenome is shorter than those of *Ventrifossa garmani* and *Bathygadus antrodes* but considerably longer than those of other Gadiform species (Table 1). Most of the genes were encoded on the heavy strand (H-strand), except for *ND6* and eight tRNA genes (Gln, Ala, Asn, Cys, Tyr, Ser(UCN), Glu, and Pro).

**Table 1:** Summary of the base composition of the mitogenomes at each codon position of the concatenated 13 protein coding genes across 11 Gadiform species.

Species	Accession number	Size(bp)	Whole genome composition							Protein-coding genes	
			A%	G%	T%	C%	A+T%	AT skew	GC skew	AT skew	GC skew
*Cetonurus globiceps*	KF751382	17137	28.10	15.60	28.10	28.20	56.20	0.0000	-0.2877	-0.0686	-0.3543
*Ventrifossa garmani*	AP008991	17230	28.17	15.85	27.80	28.18	55.97	0.0066	-0.2800	-0.0466	-0.3396
*Bathygadus antrodes*	AP008988	17596	27.59	18.78	34.94	18.69	62.53	-0.1175	0.0024	-0.2069	-0.0370
*Lota lota*	AP004412	16527	28.37	16.32	27.59	27.72	55.96	0.0139	-0.2589	-0.0304	-0.3306
*Micromesistius poutassou*	FR751401	16573	27.42	17.23	27.10	28.24	54.53	0.0059	-0.2421	-0.0455	-0.3077
*Gadus morhua*	AM489716	16654	28.06	16.79	29.56	25.59	57.62	-0.0260	-0.2076	-0.0843	-0.2593
*Arctogadus glacialis*	AM919429	16644	28.14	16.59	29.91	25.34	58.04	-0.0305	-0.2087	-0.0885	-0.2596
*Gadus chalcogrammus*	AB182305	16571	28.10	16.69	29.50	25.71	57.61	-0.0243	-0.2127	-0.0796	-0.2699
*Theragra finnmarchica*	AM489718	16571	28.10	16.70	29.51	25.69	57.61	-0.0245	-0.2121	-0.0804	-0.2689
*Boreogadus saida*	AM919428	16745	28.10	16.73	29.63	25.54	57.73	-0.0265	-0.2084	-0.0861	-0.2639
*Pollachius virens*	FR751399	16556	27.68	17.09	29.39	25.85	57.06	-0.0300	-0.2040	-0.0849	-0.2628

Among the protein-coding genes, two overlaps, *ATP8*-*ATP6* and *ND4L*-*ND4*, were detected. The transfer RNA gene pair tRNA^Ile^-tRNA^Gln^ overlaps, as well. The 32-bp fragment of O_L_, as in most vertebrates, overlaps the tRNA^Cys^ gene by 3 base pairs and is located in a cluster of five tRNA genes (WANCY region, Table 2) between tRNA^Asn^ and tRNA^Cys^. Additionally, seventeen intergenic spacers were found.

**Table 2:** Characteristic constituents of the mitochondrial genome of *C*. *globiceps*.

Gene	Position (from-to)	Intergenic Nucleotides*	Strand	Length (bp)	Start codon	Stop codon	Anticodon/ position
tRNA^Phe^	1–68		H	68			GAA/31-33
12S ribosomal RNA	69–1014	0	H	946			
tRNA^Val^	1015–1084	1	H	70			TAC/32-34
16S ribosomal RNA	1086–2738	1	H	1653			
tRNA^Leu^	2739–2811	0	H	73			TAA/36-38
ND1	2813–3784	1	H	972	ATG	TAA	
tRNA^Ile^	3789–3857	4	H	69			GAT/30-32
tRNA^Gln^	3927–3857	-1	L	71			TTG/33-35
tRNA^Met^	3927–3995	-1	H	69			CAT/31-33
ND2	3996–5045	0	H	1050	ATG	TAA	
tRNA^Trp^	5056–5126	10	H	71			TCA/33-35
tRNA^Ala^	5196–5128	1	L	69			TGC/31-33
tRNA^Asn^	5270–5198	1	L	73			GTT/34-36
tRNA^Cys^	5366–5303	32	L	64			GCA/27-29
tRNA^Tyr^	5433–5367	0	L	67			GTA/29-31
CO1	5435–6977	1	H	1543	GTG	T	
tRNA^Ser^	7054–6984	6	L	71			TGA/33-35
tRNA^Asp^	7058–7126	3	H	69			GTC/31-33
COII	7136–7820	9	H	685	ATG	T	
tRNA^Lys^	7821–7890	0	H	70			TTT/31-33
ATP8	7892–8056	1	H	165	ATG	TAA	
ATP6	8050–8732	-7	H	683	ATG	TA	
COIII	8733–9517	0	H	785	ATG	TA	
tRNA^Gly^	9518–9586	0	H	69			TCC/31-33
ND3	9587–9935	0	H	349	ATG	T	
tRNA^Arg^	9936–10004	0	H	69			TCG/31-33
ND4L	10005–10301	0	H	297	GTG	TAA	
ND4	10295–11675	-7	H	1381	ATG	T	
tRNA^His^	11676–11744	0	H	69			GTG/31-33
tRNA^Ser^	11745–11812	0	H	68			GCT/27-29
tRNA^Leu^	11814–11886	1	H	73			TAG/34-36
ND5	11887–13725	0	H	1839	ATG	AGA	
ND6	14253–13732	6	L	522	ATG	AGG	
Cytochrome b	14322–15461	68	H	1140	ATG	AGA	
tRNA^Thr^	15463–15537	1	H	75			TGT/37-39
tRNA^Pro^	15677–15608	70	L	70			TGG/32-34
tRNA^Glu^	15751–15685	7	L	67			TTC/29-31
A+T-rich region	15752–17137	0	H	1386			

* Intergenic nucleotides indicate gaps (positive values) or overlap (negative values) between consecutive genes.

The mitochondrial genome order of *C*. *globiceps* appears to have significant mitochondrial gene rearrangement relative to the typical vertebrate gene order [19]. The commonly observed tRNA^Glu^-tRNA^Thr^-tRNA^Pro^ cluster was not present in *C*. *globcipes*, instead a gene rearrangement of the cluster was observed to be tRNA^Thr^-tRNA^Pro^-tRNA^Glu^. Further comparisons revealed that the arrangement of mtDNA protein-coding genes in *C*. *globiceps* is the same as that of the species *V*. *garmani* and *Coelorinchus kishinouyei*, which belong to different subfamilies (Macrouroidinae and Trachyrincinae, respectively) [19, 41].

### Nucleotide Composition

The overall base composition of the H-strand of *C*. *globiceps* mitogenome was estimated to be 28.1% A, 28.2% C, 15.6% G, and 28.1% T, with a high A+T content (56.2%), indicating significant strand asymmetry, which is commonly observed in fish [33]. The highest A+T content was detected in the putative control region (65.6%), which is consistent with the findings of previous reports on other teleosts [42, 43]. As expected, the anti-G bias was particularly remarkable at the third codon position in 12 of the 13 protein-coding genes, while *ND6* in L-strand was C skewed. Similar GC composition patterns were also detected in other Gadiformes [44, 45].

**Fig 2:**
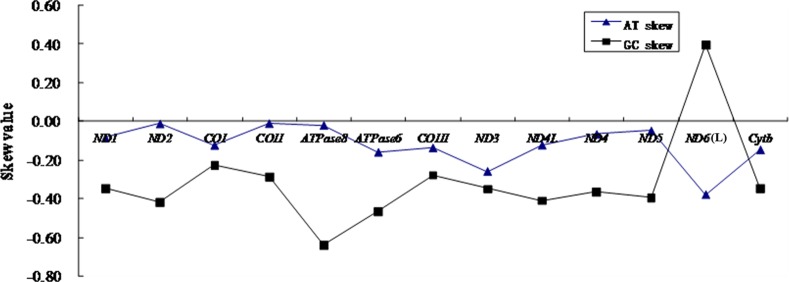
Graphical illustration showing the AT- and GC-skew in the protein coding genes of the mitochondrial genome of *C*. *globiceps*.

To further evaluate the degree of base bias, the base-skew was measured. The AT-skew and GC-skew values of the protein-coding genes of *C*. *globiceps* mtDNA are shown in Fig 2. All GC-skew and AT-skew values were negative, except for the L-strand gene *ND6*, which had a positive GC skew. This finding indicated that more Cs and Ts were present in most protein-coding genes. In the whole genome of *C*. *globiceps*, the AT-skew and GC-skew values were 0 and -0.2877, respectively (Table 1), revealing that the H-strand had equal amounts of A and T and that the overall nucleotide composition was strongly C skewed. The GC-skew and AT-skew values of protein-coding genes across 11 Gadiform species as shown in Table 1 were all negative, and it was apparent that, besides *C*. *globiceps*, the amount of Cs and Ts were more prevalent in the protein-coding genes of Gadiform species, similar to most previous observations of a bias against the use of G [46]. Notably, the two species, *C*. *globiceps* and *V*. *garmani*, which had the same rearranged gene order, were not T skewed in whole mitogenome, while most of other complete mitogenomes of gadiform species in Table 1 had a negative AT-skew value.

### Protein-Coding Genes

The 13 protein-coding genes of the *C*. *globiceps* mtDNA ranged in size from 165bp (*ATP8*) to 1839bp (*ND5*) and comprised 11,397 bp in total, accounting for 66.5% of the entire mitogenome sequence. The sizes of most protein-coding genes are similar to orthologs founds in other Gadiforme species available in the GenBank database, with the exceptions of *COI*, *COII* and *ND1*, which were shorter in length (S2 Table).

Of the 3794 codons of all protein-coding genes, excluding stop codons, the most frequently used amino acid was Leucine (18.2%), followed by Alanine (8.8%) and Threonine (7.6%). Meanwhile, the frequencies of codons encoding Cysteine and Serine possessed the lowest percentage values. Leucine was coded by six different codons, while all other amino acids were coded by either two or four different codons. Consistent with the typical strand-specific nucleotide bias in mtDNA [33], G was the least frequent nucleotide in the third position in all codons (35% C, 32% A, 27% T, 6% G). Furthermore, pyrimidines were overrepresented at the second codon position compared with purines (T+C = 68.7%), which was inferred to be a result of the hydrophobic characteristic of the proteins. Additionally, the proportion of G at the second codon position was also the lowest. The frequency of usage of the four nucleotides in the first position was nearly equal.

**Fig 3:**
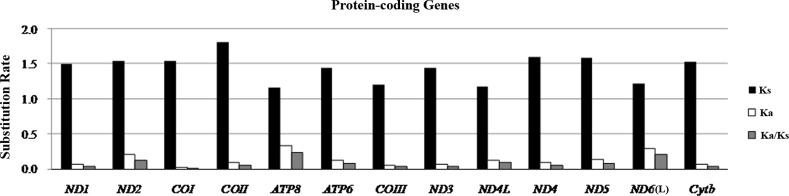
Evolutionary rates of *C*. *globiceps* mitogenome. The rate of non-synonymous substitutions (Ka), the rate of synonymous substitutions (Ks) and the ratio of the rate of non-synonymous substitutions to the rate of synonymous substitutions (Ka/Ks) for each protein-coding gene.

Methionine (ATG) is the start codon for most of the protein-coding genes, while *COI* utilizes GTG, which is also an accepted canonical mitochondrial start codon for vertebrate mitogenomes [19]. Interestingly, *ND4L* of *C*. *globiceps* begins with GTG as well, which is different than most of gadiform species, except for *V*. *garmani*. With regard to stop codons, four genes terminated with TAA, two genes end with AGA, and *ND6* ends in AGG. The other six genes had incomplete stop codons, either TA (*ATP6* and *COIII*) or T (*COI*, *COII*, *ND4* and *ND3*), which would be presumably completed as entire stop codon (TAA) via post-transcriptional polyadenylation. In all 13 protein-coding genes, the average Ka/Ks ratio varied from 0.0177 (*COI*) to 0.2462 (*ATP8*) and was lower than 0.5 for all other genes (Fig 3). As is reported, two nonadaptive forces, random genetic drift and mutation pressure define the fundamental features of genome evolution. However, functional constraint imposes burden on mutation [47]. Therefore, the mutation-associated disadvantages are difficult to establish under purifying selection. The selection processes maintain long-term stability of the biological structure. Our result of the Ka/Ks ratio indicated that the various functional genes evolved under strong purifying selection which means natural selection against deleterious mutations with negative selective coefficients [48]. The selection pressures differed among genes [47], and they are likely to evolve in different ways. Additionally, *ATP8* and *ND6* had the highest ratios, indicating that the selection pressures were independent of which strand the gene was located.

**Fig 4:**
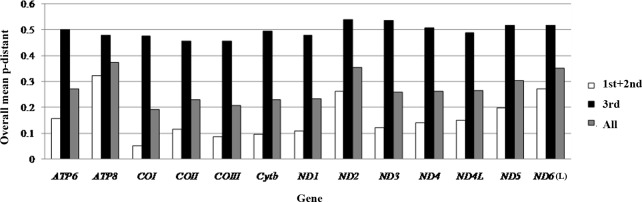
Overall mean p-genetic distance of six Macrouridae species for each of 13 protein genes. They were calculated based on the first and second nucleotide positions and on the third nucleotide position of amino acid codons, and on the full sequence among six Macrouridae species, respectively.

The conservation of mtDNA genes was evaluated based on the overall p-genetic distance among six Macrouridae species, including *C*. *globiceps*, *V*. *garmani*, *Trachyrincus murrayi*, *Squalogadus modificatus*, *C*. *kishinouyei*, and *Arctogadus glacialis* [49]. Of the 13 protein-coding genes, the *COI* gene has the lowest overall p-genetic distance (0.051) among species, and the *ATP8* gene has the highest value (0.321) based on data of the first and second nucleotides of codons. According to full-length sequence comparisons for each gene, *ATP8* also has the highest value (0.374), and *COI* has the lowest value (0.193). Base on these results, it can be found that *ATP8* likely has the fastest evolutionary rate among Macrouridae species, while *COI* has the lowest rate. For the third nucleotide, all genes have a high overall p-genetic distance value. As is the case for other fish, most of the differences in the mtDNA protein-coding genes occurred at the third codon position [34] (Fig 4).

### Transfer and Ribosomal RNA Genes

The *C*. *globiceps* mitogenome contained 22 tRNA genes that are typically found in most metazoan mitogenomes. They varied in size from 64 bp (tRNA^Cys^) to 75 bp (tRNA^Thr^), and the anticodons were identical to those reported in other vertebrates. Of these tRNAs, two were determined to be for serine (UCN and AGY), and two were for leucine (UUR and CUN). All of the22 tRNA genes, with the exception of tRNA^Ser (AGY)^, could be folded into canonical cloverleaf secondary structures using the tRNA Scan-SE software [26]. As in vertebrates in general, the secondary structure of tRNA^Ser(AGY)^, predicted with ARWEN [50], lacks a discernible dihydrouridine (DHU) stem (S1 Fig).

A total of 55 mismatched base pairs were found in the postulated secondary structures of 21 tRNAs, with 24 in the amino acid acceptor stems, 12 in the DHU stems, 8 in the pseudouridine (TΨC) stems and 11 in the anticodon stems. These unmatched base pairs showed significant bias, with 33 G-U pairs, 9 A-C pairs, 4 U-U pairs, 3 A-A pairs, 2 A-G pairs, 2 U-C pairs and 2 C-C pairs. The mismatches can be corrected via manual editing [51].

The 12S and 16S rRNA genes were 946 and 1653 bp, respectively. They were both on the sense strand and were located between tRNA^Phe^ and tRNA^Leu(UUR)^. They were separated by the tRNA^Val^, as is the case in most vertebrates [19]. The base composition of the 12S ribosomal gene is 30.87% A, 21.56% G, 22.20% T and 25.37% C, with an A+C content of 56.24%. The 16S RNA composition is 34.2% A, 19.36% G, 21.84% T and 24.56% C, A+C accounting for 56.04% of the content. It indicated an A+C-rich trend as in other bony fish [52].

### Non-Coding Regions

**Fig 5:**
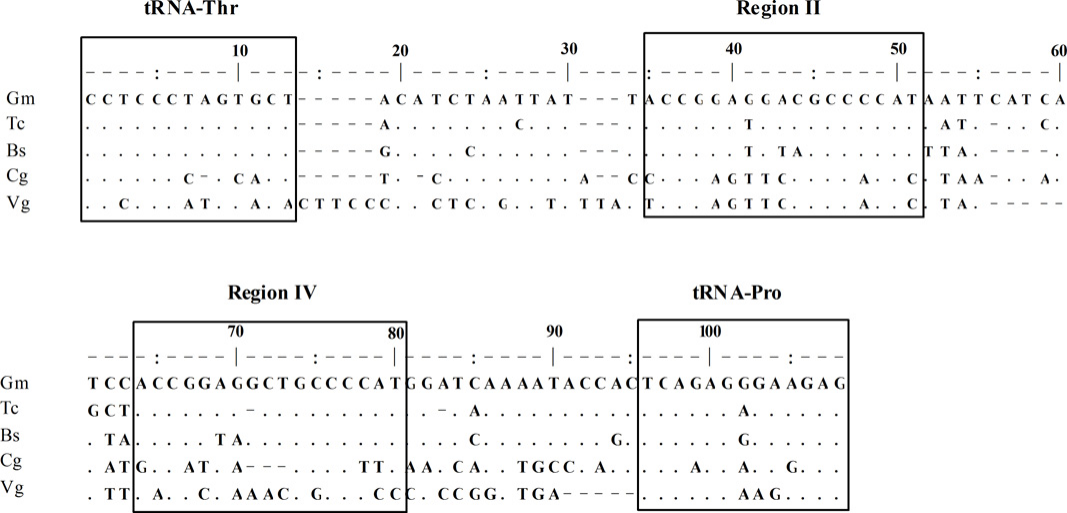
Intergenic T-P spacer sequence of five Gadiform species. Gm: *Gadus morhua*, Tc: *Theragra chalcogramma*, Bs: *Boreogadus saida*, Cg: *Cetonurus globiceps*, Vg: *Ventrifossa garmani*. Dots indicate identical positions and dashes indicate deletions. Conserved regions and tRNAs are marked by boxes.

A codfish-unique T-P spacer, 70bp in size, was also observed in *C*. *Globiceps* [20, 40]. After sequence alignment with the published corresponding sequences of *Gadus morhua (Gm)*, *Theragra chalcogramma* (Tc) and *Boreogadus saida* (Bs), the spacer was revealed to be similar to that in other codfishes thus the sequence was divided into five regions (regions I-V, Fig 5), following the findings of Bakke and colleagues [40]. As previously observed, region II of the T-P spacer in *C*. *globiceps* was found to be the most conserved region in terms of the sequence as well as the placement, while other regions showed great variability (Fig 5). The T-P intergenic spacer being found in all species of the family Gadidae is indicative of its evolution before the family began to diversify and of its evolutionary stability over time [20, 40]. It has become clear, regarding the evolution of mtDNA genomes, that genomic size is usually minimized under strong purifying selection [53]. Thus, the presence of conserved non-coding motifs in *C*. *globiceps* as well as in other codfish suggests that they are evolutionarily homologous and supports a conservation of universal important functions, such as mitochondrial replication and/or transcription [20]. Notably, it was interesting that the highest sequence similarities of regionII appeared in *C*. *globiceps* and *V*. *garmani*, which belong to different subfamilies of Macrouridae. Considering that it is unlikely for different species to acquire the same element by chance, we propose that different species of Macrouridae might share similar molecular characteristics of the ancestral T-P spacer. These similarities may reflect the phylogenetic relationship between the species.

**Fig 6:**
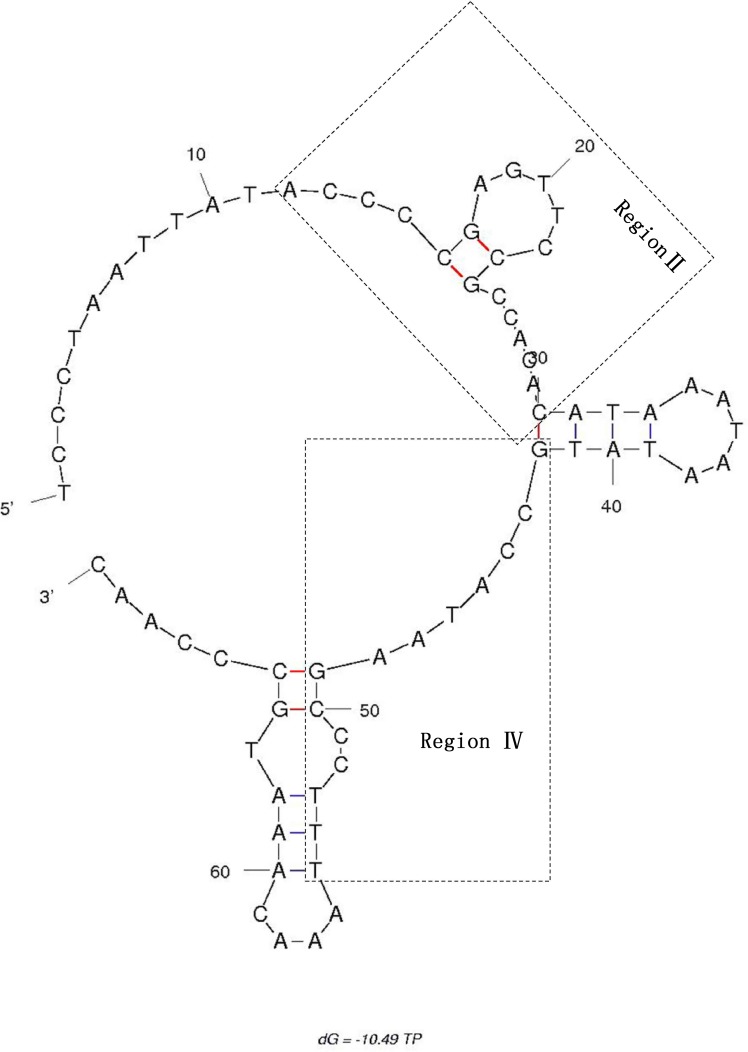
Secondary structure prediction for the T-P spacer DNA sequence of *C*. *globiceps*.

The secondary structure analysis indicated that the conserved region II as well as region III and regions IV-V formed a relatively stable stem-and-loop structure, with a free energy of -10.49 kcal/mol, which might conceivably protect the spacer from excision during the single-stranded phase of mtDNA replication (Fig 6). However, these non-coding sequences combined with the gene rearrangements of mitogenome sequences in the region from *ND6* to tRNA^Pro^ in *C*. *globiceps* and other Gadiformes appear to argue against the T-P spacer being an insertion from nuclear sequences or from another mtDNA region [20, 40]. It may represent vestiges of partial loss of certain duplicated portion occurring during sequence duplication. The duplication-random loss model thus seems to be the preferred hypothesis [20, 40].

**Fig 7:**
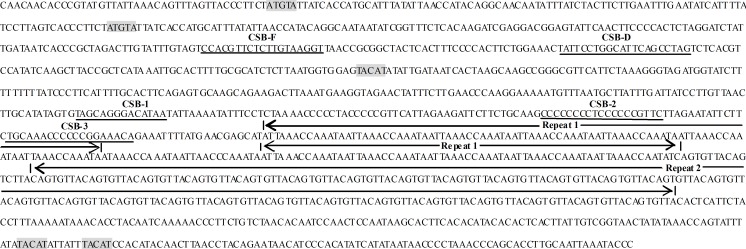
Features present in the putative control region of *C*. *globiceps* mitogenome. The conserved motifs ATGTA and its complement TACAT which may form thermostable hairpin structure are shaded. The conserved sequence blocks containing CSB-F, CSB-D, CSB-1,CSB-2 and CSB-3 are underlined and labeled by name. The arrows above the nucleotides indicate the tandem repeats.

Similar to that in most vertebrates, the control region of *C*. *globiceps* was adjacent to the 5’ portion of tRNA^Phe^ [33, 46,54]. The control regions of teleost fish contain a number of conserved sequence blocks (CSBs), which may play important roles in mitochondrial metabolism [14]. CSB-D, -E and–F are found typically present in central conserved domain of control region of teleost fish and CSB-1, CSB-2 and CSB-3 present in the conserved sequence block domain [14]. By comparing with the recognition sites in teleost species, several conserved sequence blocks containing CSB-F, CSB-D, CSB-1,CSB-2 and CSB-3 have been identified (Fig 7) [45]. These sequence elements are thought to function as proper regulatory sites as well as recognition sites for transcription primer strand synthesis and for replication priming [34]. Further study into the mechanism of mtDNA transcription and replication is thus warranted. Consistent with other Gadiformes, the GTGGG-box, which is the typical characteristic of CSB-E, was absent in *C*. *globiceps*, while this motif is commonly found in teleosts [33, 54]. Notably, tandem repeats are indicated in the 3' portion of CR, and it harbors two types of tandem repeats comprising a 13 bp repeat (ATTAAACCAAATA) and 8 bp repeat (CAGTGTTA) unit, named Repeat 1 and Repeat 2 respectively and shown in Fig 7. No similar repetitive motif is present in other gadiform species, except *V*. *garmani*, suggesting that the duplication events occurred after family diversification. Nevertheless, more information is needed to provide a useful model system to study how these repeat clusters evolved.

### Phylogenetic Analyses

**Fig 8:**
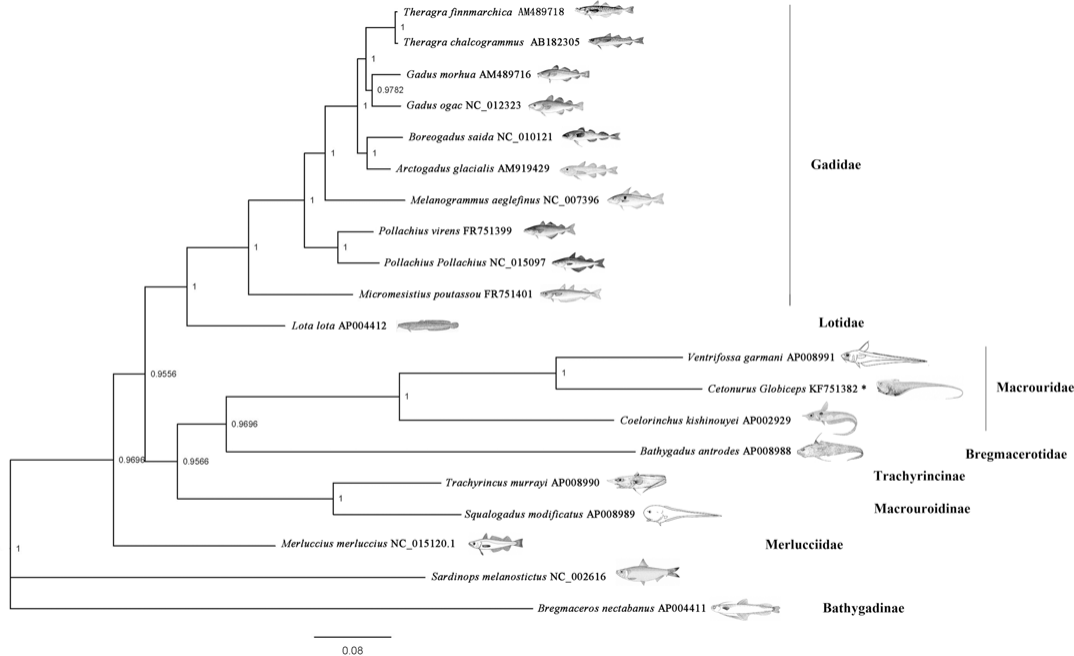
Phylogenetic tree of Gadiform species reconstructed from concatenated DNA sequences of mitochondrial protein-coding genes. Twelve mitochondrial protein-coding genes (with the exception of *ND6*) were used for the phylogenetic tree, which was produced by Bayesian inferences (BI). *Sardinops melanostictus* was used as the outgroup. Bayesian posterior probability are shown orderly on the nodes. The asterisk indicates the sequence generated in this study.

Gadiformes have been typically positioned in the center of Paracanthopterygii [10]. However, the systematic organization within this order represents a problematic issue. Endo, in 2002, reported a comprehensive classification of Gadiformes and formed the basis for the current Gadiformes classification system [10]. However, the results still reflected several unresolved relationships [41]. Following a number of molecular studies conducted to resolve the phylogenetic relationships at the genus level [55, 56], the most recent work, carried out by Roa-Varon and colleague, made great progress in confirming the monophyly of most of the proposed families for Gadiformes. However, taxon samples remained limited [41]. In this study, the phylogenetic position of *C*. *globiceps*, based on the 12 concatenated protein-coding genes of mitochondria, was first analyzed. The accession numbers for the mitochondrial genome sequences of the 19 species utilized in this study were shown in Fig 8. Different methods (BI, ML and NJ) generated similar tree topologies, and most of the nodes were statistically supported by high bootstrap and posterior probability values. Only the Bayesian tree is shown in Fig 8. The phylogenetic trees included 19 Gadiform species, representing 7 families and 16 genera, and revealed two main clades, namely the suborders Macrouroidei and Gadoidei, following the taxonomic system proposed by Roa-Varon and colleague [41]. Four Macrouridae species including *C*. *globiceps*, *Bathygadus antrodes*, *Coelorinchus kishinouyei* and *Ventrifossa garmani* formed a monophyletic group. Our findings suggests that the genetically closest relationship exists between *C*. *globiceps* and *V*. *garmani*. The two species were shown to be in the most evolved clade (Fig 8.). Combined with the previously mentioned, unexpected motifs in the control regions of *C*. *globiceps* and *V*. *garmani*, which were absent in other gadiform fish, it is reasonable to assume that these two species might have separated recently. Furthermore, the hydraulic pressure of the marine environments plays a pivotal role in the speciation process of fish that geographically evolve from shallow environments to the deep sea [57, 58]. *V*. *garmani* was distributed at a depth ranging from 200–720 m, which is much shallower than that of *C*. *globiceps*. These clues may indicate that the control region evolved before *C*. *globiceps* had adapted to the deep-water environment. However, further investigation such as heterologous assays *in vitro* or hybridization experiments is needed to determine whether the structure of the non-coding region was important in adaptation and acclimation to the deep sea environment.

Notably, *M*. *merluccius*, a member of the family Merlucciidae, was placed as a single branch, separate from all other Gadiform species selected in our study. Although many other studies have found the same result, Roa-Varon identified Merlucciidae within Gadoidei with a high level of support [41]. It was found that the pattern of gene rearrangement in the mitochondrial DNA of *M*. *merluccius* differed from that of other Gadiform species [19, 32], and it seems to be correlated between rearrangement rates and nucleotide substitution frequency. Thus, more evidence is required for phylogenetic placement, which appears ambiguous, to carefully clarify or re-evaluate the phylogenetic relationships among these major gadiform clades. The topologies of the three phylogenetic trees in our study, similar to previous studies, failed to reach a consensus on the relationships between *B*. *nectabanus* and other species. *T*. *murrayi* and *S*. *modificatus*, which belong to Trachyrincinae, were paraphyletic with Macrouridae, which is contradictory to data by Roa-Varon but consistent with most previous studies [41]. More evidence is required to elucidate the phylogenetic relationships that appear to be ambiguous.

## Conclusion

The present study determined the complete mtDNA sequences of *C*. *globiceps* for the first time. The mtDNA sequence is 17,137bp in length and contains the typical set of 13protein-coding genes, two rRNA genes, 22 tRNA genes and two major non-coding regions (the origin of light strand replication and control region). Additionally, a 70bp T-P intergenic spacer that is unique to Gadiformes was observed. Similarly to two other Macrouridae species, significant mitochondrial gene rearrangement relative to the gene arrangement of typical teleosts was found. Phylogenetic analysis using 12 concatenated mitochondrial protein-coding genes indicated that *C*. *globiceps* and *V*. *garmani* were most closely related. Some relationships that differed from previous studies were also observed. These relationships could be artifacts from the very narrow taxon sampling conducted for this study. The complete mitogenomic information presented here is expected to enrich our knowledge on the mitogenomes of the genus *Cetonurus* and to provide important data for further studies on population genetics and evolutionary biology of Gadiforms.

## Supporting Information

S1 Fig Putative secondary structure of the 22 tRNAs identified in the mitochondrial genome of *Cetonurus globiceps*. The tRNAs are labeled with the abbreviations of their corresponding amino acids.
(DOC)

S1 Table Main PCR primers used in the analysis of *Cetonurus globiceps* mitochondrial genome. (DOCX)

S2 Table The sizes of 13 protein-coding genes of *Cetonurus globiceps* and 14 other Gadiforme species available in the GenBank database. (DOCX)
