# Supplementary figures and images for: Characterization of the Complete Mitochondrial Genome Sequence of the Globose Head Whiptail Cetonurus globiceps (Gadiformes: Macrouridae) and Its Phylogenetic Analysis
Source: PLoS One. 2016 Apr 19;11(4):e0153666. doi: 10.1371/journal.pone.0153666 (PMC4836748; doi:10.1371/journal.pone.0153666)

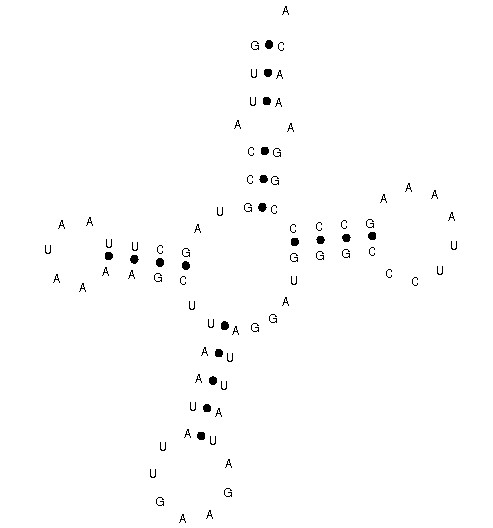

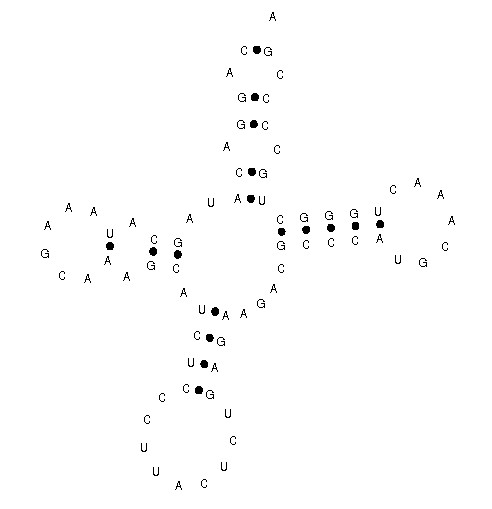

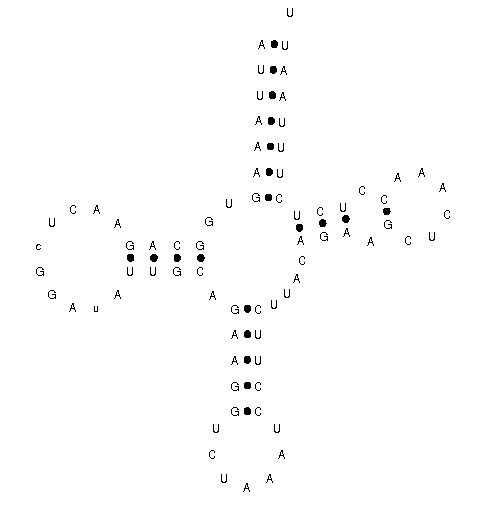


Phe

Val

Leu-UUR


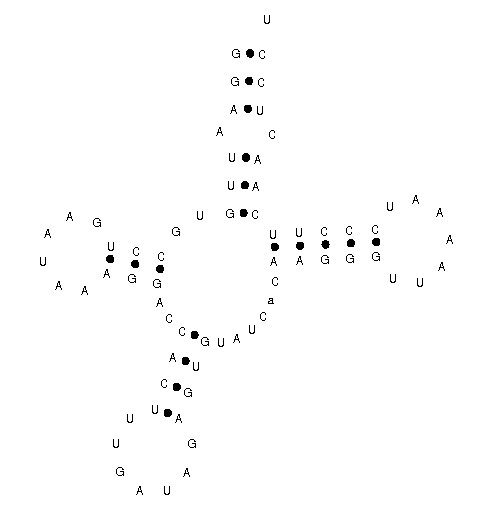

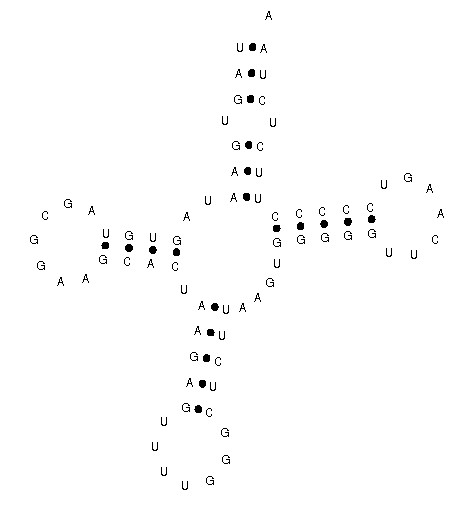

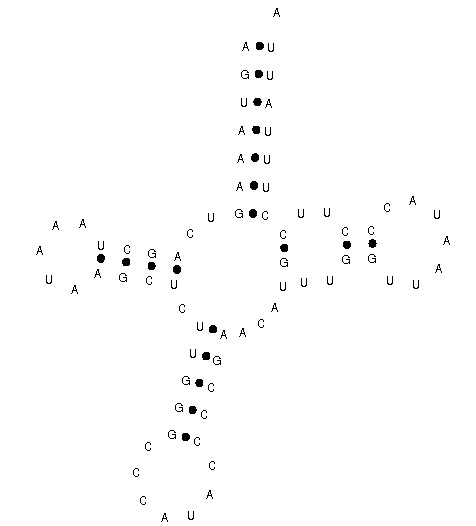


Ile

Gln

Met


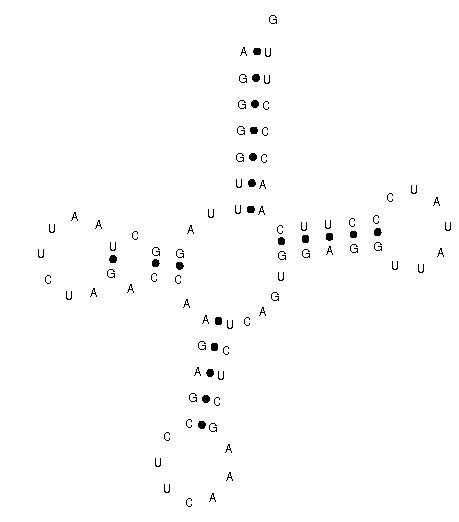

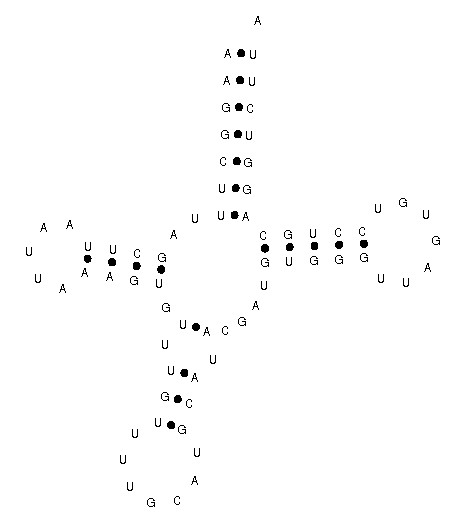

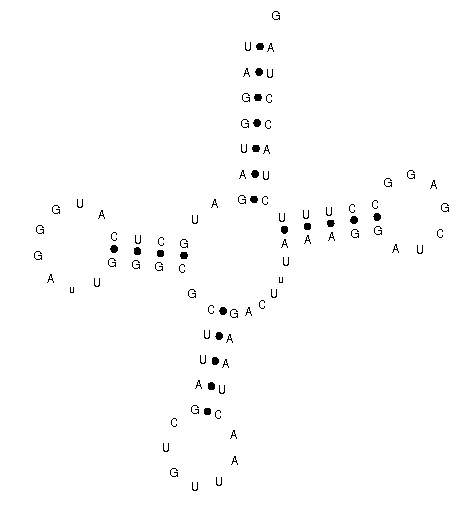


Trp

Ala

Asn


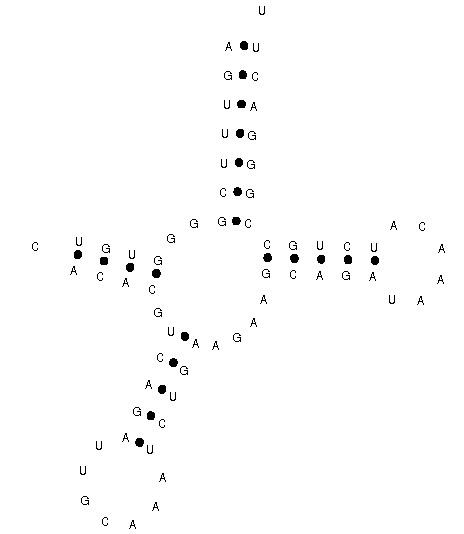

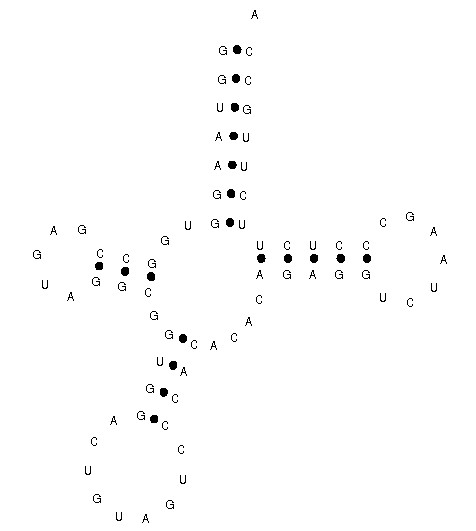

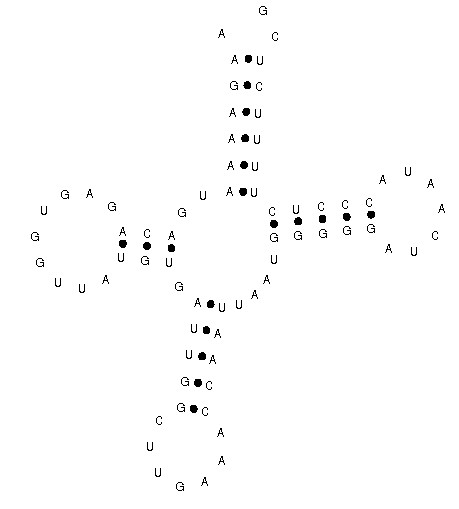


Cys

Tyr

Ser-UCN


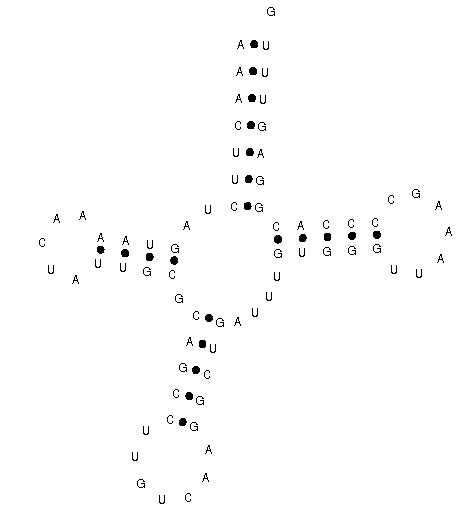

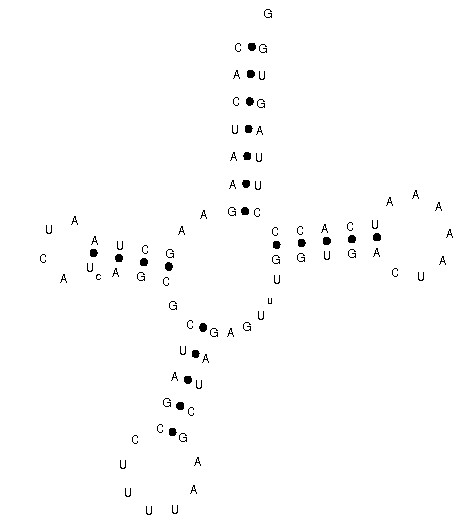

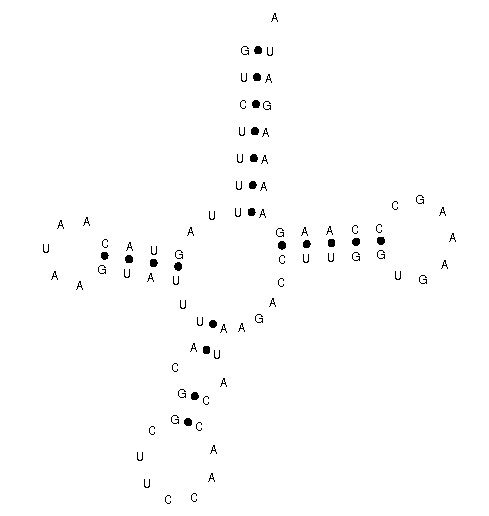


Asp

Lys

Gly


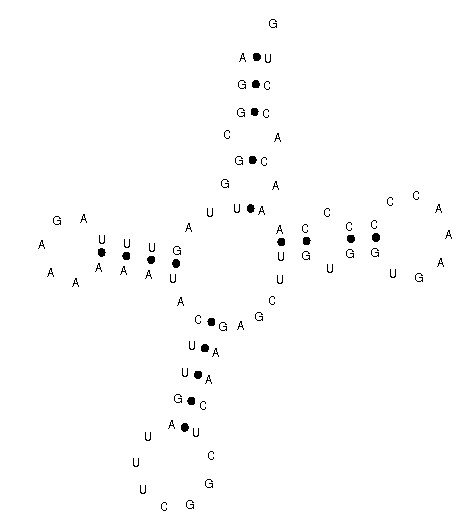

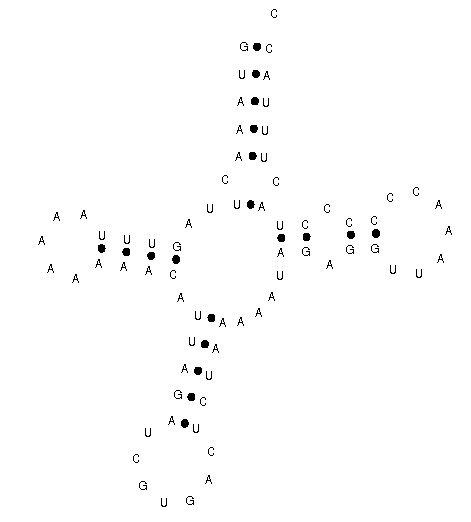

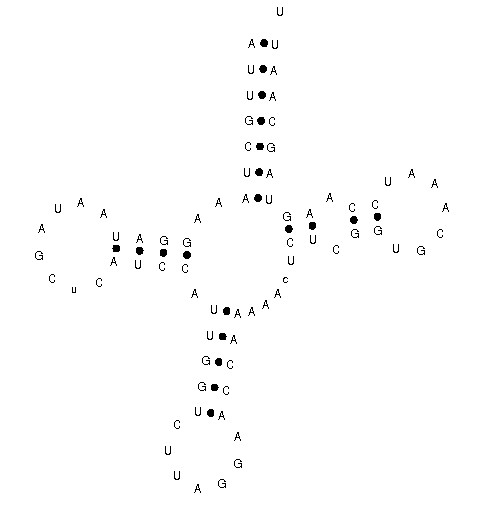

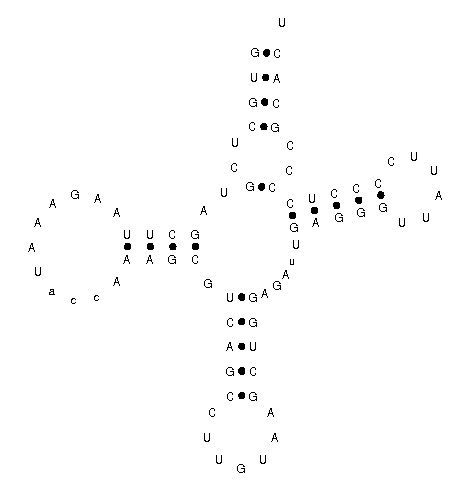

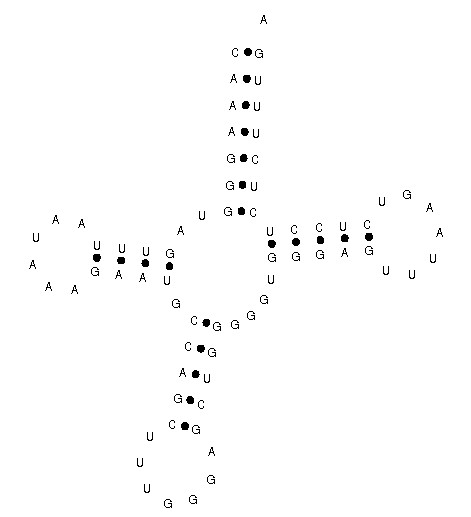

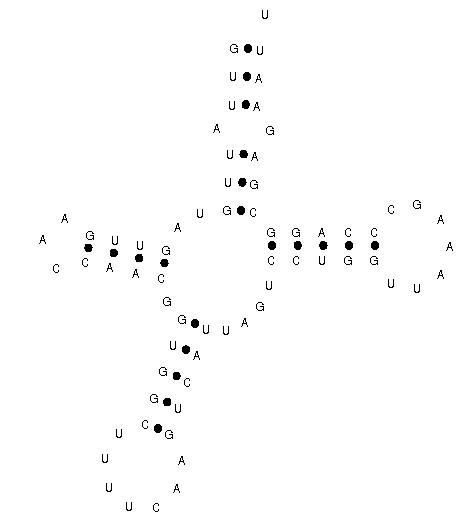


Arg

His

Leu-CUN

Thr

Pro

Glu


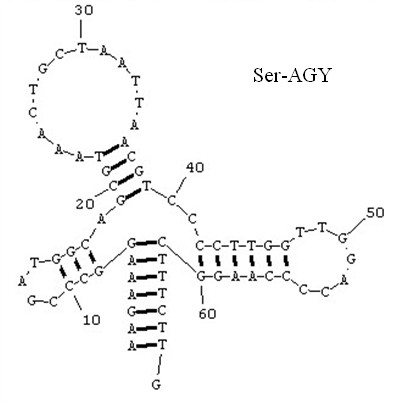

Supplement: S1 Fig — The tRNAs are labeled with the abbreviations of their corresponding amino acids. (DOC) [file pone.0153666.s001.doc]
