# Supplementary material for: Characterization of the Complete Mitochondrial Genome Sequence of the Globose Head Whiptail Cetonurus globiceps (Gadiformes: Macrouridae) and Its Phylogenetic Analysis
Source: PLoS One. 2016 Apr 19;11(4):e0153666. doi: 10.1371/journal.pone.0153666 (PMC4836748; doi:10.1371/journal.pone.0153666)
Supplement: S1 Table — (DOCX) [file pone.0153666.s002.docx]

**S1 Table Main PCR primers used in the analysis of *C. globiceps* mitochondrial genome**

| code | Forward primer name | Forward primer sequence  (5’-3’) | Reverse primer name | Reverse primer sequence  (5’-3’) | product length  (bp) | Annealing  temperature(℃) |
| --- | --- | --- | --- | --- | --- | --- |
| 1 | L1-12S | GACAGACAACGGCGTAAAGCG | H1-ND2 | CCTCAAAGGATAATGCCTGCTC | 3786 | 50 |
| 2 | L2-MET | GGCCCATACCCCGAACATGT | H2-COII | TCTATTASGGGWGATGCYGCGTCT | 3163 | 53 |
| 3 | L3-SER | TGGTTTCAAGCCARTCACAT | H3-ARG | CTTGAGCCGAAATCAAGTAT | 2902 | 60 |
| 4 | L4-ND3 | GAAATCGCACTCTTACTACCT | H4-ND4L | GTTAGTCAGATGGCGGGAAT | 565 | 50 |
| 5 | L5-ARG | ACTTGATTTCGGCTCAAGAG | H5-GLU | ACGGTGGTTTTTCAAGTCAT | 5710 | 60 |
| 6 | L6-THR | CAACTCAAAGAGAGGAGACT | H6-CR | TTGAATACTCCGTCCTCGAT | 273 | 50 |
| 7 | L7-GLU | GACTTGAAAAACCACCGTTG | H7-12S | GTGGGGTATCTAATCCCAGT | 1853 | 55 |
