# Supplementary material for: Characterization of the Complete Mitochondrial Genome Sequence of the Globose Head Whiptail Cetonurus globiceps (Gadiformes: Macrouridae) and Its Phylogenetic Analysis
Source: PLoS One. 2016 Apr 19;11(4):e0153666. doi: 10.1371/journal.pone.0153666 (PMC4836748; doi:10.1371/journal.pone.0153666)
Supplement: S2 Table — (DOCX) [file pone.0153666.s003.docx]

|  | *COI* | *COII* | *ATP8* | *ATP6* | *COIII* | *ND3* | *ND4L* | *ND4* | *ND5* | *ND6* | *Cyt b* | *ND1* | *ND2* |
| --- | --- | --- | --- | --- | --- | --- | --- | --- | --- | --- | --- | --- | --- |
| *Cetonurus globiceps* | 1543 | 685 | 165 | 683 | 785 | 349 | 297 | 1381 | 1839 | 522 | 1140 | 972 | 1050 |
| *Arctogadus glacialis* | 1551 | 691 | 168 | 683 | 786 | 349 | 297 | 1381 | 1839 | 522 | 1141 | 975 | 1045 |
| *Bathygadus antrodes* | 1551 | 691 | 162 | 683 | 785 | 349 | 297 | 1381 | 1839 | 519 | 1141 | 975 | 1042 |
| *Boreogadus saida* | 1551 | 691 | 168 | 683 | 786 | 349 | 297 | 1381 | 1839 | 522 | 1141 | 975 | 1045 |
| *Gadus chalcogrammus* | 1551 | 699 | 168 | 684 | 786 | 351 | 297 | 1386 | 1839 | 522 | 1611 | 975 | 1047 |
| *Gadus morhua* | 1551 | 699 | 168 | 684 | 786 | 351 | 297 | 1386 | 1839 | 522 | 1140 | 975 | 1047 |
| *Lota lota* | 1551 | 691 | 168 | 683 | 785 | 349 | 297 | 1381 | 1839 | 522 | 1141 | 975 | 1045 |
| *Micromesistius poutassou* | 1551 | 691 | 168 | 682 | 786 | 349 | 297 | 1381 | 1839 | 522 | 1141 | 975 | 1045 |
| *Pollachius virens* | 1551 | 691 | 168 | 682 | 786 | 349 | 297 | 1381 | 1839 | 522 | 1141 | 975 | 1045 |
| *Theragra finnmarchica* | 1551 | 691 | 168 | 684 | 786 | 351 | 297 | 1386 | 1839 | 520 | 1141 | 975 | 1047 |
| *Coelorinchus kishinouyei* | 1546 | 685 | 168 | 683 | 785 | 349 | 297 | 1381 | 1845 | 522 | 1140 | 972 | 1047 |
| *Ventrifossa garmani* | 1548 | 685 | 165 | 683 | 785 | 349 | 297 | 1381 | 1839 | 522 | 1140 | 972 | 1050 |
| *Ventrifossa garmani* | 1548 | 685 | 165 | 683 | 785 | 349 | 297 | 1381 | 1839 | 522 | 1140 | 972 | 1050 |
| *Coelorinchus kishinouyei* | 1546 | 685 | 168 | 683 | 785 | 349 | 297 | 1381 | 1845 | 522 | 1140 | 972 | 1047 |
| *Merluccius merluccius* | 1551 | 691 | 168 | 684 | 786 | 349 | 297 | 1381 | 1839 | 564 | 1141 | 975 | 1045 |

**S2 Table The sizes of 13 protein-coding genes of *Cetonurus globiceps* and 14 other Gadiforme species available in the GenBank database**
